# Cognitive effects of methamphetamine and amphetamine withdrawal in rodents: a systematic review

**DOI:** 10.3389/fpsyg.2026.1729722

**Published:** 2026-03-26

**Authors:** Reshiika Poorvii, Isa Naina Mohamed, Mohamad Fairuz Yahaya, Norazrina Azmi, Teoh Seong Lin, Rashidi Mohamed Pakri Mohamed, Azizah Ugusman, Prem Kumar Shanmugam, Jaya Kumar

**Affiliations:** 1 Department of Physiology, Faculty of Medicine, Universiti Kebangsaan Malaysia, Kuala Lumpur, Malaysia; 2 Department of Pharmacology, Faculty of Medicine, Universiti Kebangsaan Malaysia, Kuala Lumpur, Malaysia; 3 Department of Anatomy, Faculty of Medicine, Universiti Kebangsaan Malaysia, Kuala Lumpur, Malaysia; 4 Centre for Drug and Herbal Development, Faculty of Pharmacy, Universiti Kebangsaan Malaysia, Kuala Lumpur, Malaysia; 5 Department of Family Medicine, Faculty of Medicine, Universiti Kebangsaan Malaysia, Kuala Lumpur, Malaysia; 6 Manchester Metropolitan University, Kota Kinabalu, Malaysia

**Keywords:** abstinence, amphetamine, cessation, cognition, executive function, learn, memory, methamphetamine

## Abstract

**Background:**

Methamphetamine (METH) and amphetamine (AMPH) are widely misused psychostimulants that induce enduring alterations in brain function and behavior, including cognitive impairment. To date, literature in this area has not been sufficiently reviewed and summarized to account for how methodological variables, such as dosage and administration contingency, influence cognitive outcomes.

**Objective:**

This systematic review evaluates the effects of METH and AMPH withdrawal on rodent cognition, with particular attention to how outcomes are influenced by dose, sex, strain, and withdrawal duration.

**Methods:**

A systematic search of Web of Science, PubMed, Scopus, and OVID was conducted following PRISMA 2020 guidelines. Eligible studies were full-text, English-language articles assessing cognition in rodents after withdrawal from METH or AMPH. Risk of bias was evaluated using SYRCLE’s tool. Ultimately, 37 original articles published between 1971 and 2025 were included in this review.

**Results:**

Withdrawal impaired recognition and non-spatial working memory (novel object recognition and temporal order) as well as spatial working memory (Morris Water Maze, object placement recognition, Y-maze, radial arm maze, and T-maze). Outcomes varied according to dose, withdrawal duration, sex, and strain. Some studies reported partial recovery or even enhanced reversal learning with prolonged abstinence. Extended-access and high-dose regimens produced more persistent deficits, with females generally more vulnerable than males. Locomotor findings were inconsistent: some studies reported hypoactivity or impaired motor coordination, while others observed no change.

**Conclusion:**

Rodent evidence indicates that METH and AMPH withdrawal most reliably disrupt recognition and working memory, with less consistent effects on spatial learning and locomotion. Standardization of dosing regimens, withdrawal periods, and behavioral tasks is needed to improve reproducibility and enhance translational relevance to human addiction research.

## 1 Introduction

Methamphetamine (METH) and amphetamine (AMPH) are both classified under amphetamine-type stimulants (ATS) and are widely misused worldwide. Both compounds produce broadly similar psychoactive effects; although in humans, METH is generally more potent and is associated with greater risks to individual health and wellbeing (UNODC, 2025). The clinical application of these stimulants requires careful consideration of the balance between therapeutic benefits and the potential for adverse effects and recreational misuse (Heal et al., 2013). For example, Adderall, which contains mixed l-AMPH and d-AMPH salts, is commonly prescribed for the treatment of attention-deficit/hyperactivity disorder (ADHD), narcolepsy, and, less commonly, obesity (Paz-Ramos et al., 2023).

In 2020, approximately 30 million people, representing 0.4% of the global population, reported using METH, with the highest prevalence observed in East and Southeast Asia and the highest proportional use reported in North America (United Nations Office on Drugs and Crime Research, 2022). Recent global data across 47 countries further highlights this public health challenge among younger populations, revealing a 4.05% prevalence of amphetamine or methamphetamine use among school-going adolescents aged 12–15 years, with the highest regional rates observed in Africa (4.34%) (Son et al., 2025).

In the mammalian brain, METH is a potent central nervous system stimulant that disrupts the release and reuptake of monoamines, particularly dopamine, norepinephrine, and epinephrine (Abbruscato and Trippier, 2018), resulting in a wide range of behavioral and cognitive effects. Crystalline METH represents the most potent form and is strongly associated with dependence, often leading users to escalate dosages and engage in prolonged use (McKetin et al., 2006; European Monitoring Centre for Drugs and Drug Addiction, 2022). Similar to METH, AMPH also acts as a potent central nervous system stimulant, disrupting monoamine pathways (dopamine, norepinephrine, and epinephrine) to cause similar behavioral and cognitive effects (Paz-Ramos et al., 2023). While crystalline METH is generally more potent, AMPH also exhibits a strong association with dependency, often leading to dosage escalation and prolonged use (Siefried et al., 2020). Withdrawal from stimulants like METH and AMPH is accompanied by psychological and physiological distress such as exhaustion, sleep disturbance, increased appetite, depression, mood swings, physical discomfort, cognitive dullness, anhedonia and craving for drug (Substance Abuse and Mental Health Services Administration [SAMHSA], 2021). This is a clinical syndrome that manifests when the body and brain attempt to readjust to functioning without these substances after a period of prolonged use.

In rodent models, the chronic administration of stimulants, such as METH precipitates a complex cascade of neurobiological maladaptation that underpin subsequent cognitive decline. Central to these changes is the profound disruption of monoaminergic systems; chronic exposure leads to the depletion of dopamine and serotonin, alongside a significant reduction in their respective transporter densities within the striatum and prefrontal cortex (Sepulveda et al., 2021; Li and Shoptaw, 2023). Beyond neurotransmitter depletion, AMPH and METH triggers robust neuroinflammatory responses, characterized by microglial activation and the release of pro-inflammatory cytokines, which exacerbate neuronal damage and impair synaptic plasticity (Shi et al., 2022; Cabrera et al., 2022). These insults are often accompanied by oxidative stress and mitochondrial dysfunction (Chen et al., 2022), further compromising cellular integrity. At the synaptic level, withdrawal disrupts glutamatergic signaling, evidenced by altered expression of AMPA receptor subunits such as GluA1, and affects cholinergic modulation, both of which are critical for executive function and memory (Memos et al., 2023; Ferrucci et al., 2019; Peleg-Raibstein et al., 2009).

Users of stimulants, particularly those who engage in high-dose binge administration over prolonged periods, exhibit persistent neuroadaptive changes and neurotoxic damage to central dopaminergic and serotonergic systems, especially within the frontostriatal pathway (Paz-Ramos et al., 2023). Low-to-moderate stimulant use has been reported to enhance selective attention and vigilance (Silber et al., 2006) and to reduce psychomotor response time in humans (Narayan et al., 2021; Hayley et al., 2023). However, these cognitive-enhancing effects are variable and tend to diminish with higher doses or repeated drug exposure (Hayley et al., 2023).

While several recent comprehensive reviews have addressed cognitive deficits associated with stimulant use (Bernheim et al., 2016; Tamijani et al., 2023; Sim et al., 2022, Khan et al., 2025;), a systematic comparison focusing specifically on the withdrawal-induced impairments of both METH and AMPH in rodent models remains a novel contribution to the field. Therefore, this review aims to systematically examine the cognitive impairments induced by METH and AMPH specifically during the withdrawal period in rodents. This systematic review specifically focuses on rodent models to minimize the confounding variables often present in human clinical studies, such as poly-drug use, varying socioeconomic factors, and pre-existing psychiatric conditions. Furthermore, pre-clinical research allow for the precise control of drug dosage, administration contingency, and withdrawal duration, providing a clearer understanding of the direct neurobiological impacts of stimulants on cognitive domains. In the context of this review, “withdrawal” is defined as the physiological and behavioral state following the cessation of drug exposure. This term is used as a broad descriptor encompassing both the acute phase of drug removal and the subsequent period of protracted abstinence to align with the transition from drug-dependence to a drug-free state, during which cognitive deficits manifest or persist.

## 2 Methodology

### 2.1 Search strategy

This systematic review was conducted in accordance with the Preferred Reporting Items for Systematic Reviews and Meta-Analyses (PRISMA) guidelines, originally published in 2009 and updated in 2020, to ensure transparent and comprehensive reporting (Page et al., 2021a, 2021b). Studies were retrieved from four electronic databases: Web of Science, PubMed, Scopus, and OVID, with no date restrictions applied. The search was performed on 1st December 2025. The search strategy incorporated the following keywords and Boolean operators: (amphetamine OR methamphetamine) AND (abstinence OR withdrawal OR cessation) AND (cognit* OR learn* OR memory OR executive). To ensure a comprehensive analysis of the timeline following drug discontinuation, the search strategy utilized the terms “withdrawal,” “abstinence,” and “cessation.” These terms were chosen to capture the full temporal spectrum of cognitive impairment, ranging from immediate acute effects (within 24 h) to long-term abstinence periods.

### 2.2 Inclusion criteria

Eligible studies were full-length articles published in English that investigated the cognitive effects of METH or AMPH withdrawal in rodent models.

### 2.3 Exclusion criteria

During title screening, human studies, reviews, case series, books, letters to the editor, cell culture studies, and conference abstracts were excluded. In the full-text screening, studies were excluded based on the following criteria: (1) studies conducted in humans or animals other than rodents; (2) absence of a defined withdrawal or abstinence period prior to testing; (3) lack of behavioral testing or use of tasks not directly related to cognitive assessment (e.g., studies focused solely on locomotor activity or drug-seeking/reinstatement); (4) experimental designs primarily examining drug priming or those lacking a stimulant-only (vehicle-treated) control group to serve as a baseline for cognitive impairment; and (5) failure to report primary data or statistical results for the cognitive performance of the stimulant-exposed groups.

### 2.4 Risk of bias

Risk of bias was assessed using SYRCLE’s risk of bias tool for animal studies. The types of bias evaluated included selection, performance, detection, attrition, reporting, and other sources of bias. This tool consists of 10 domains: sequence generation (D1), baseline characteristics (D2), allocation concealment (D3), random housing (D4), performance bias blinding (D5), random outcome assessment (D6), detection bias blinding (D7), incomplete outcome data (D8), selective outcome reporting (D9), and other sources of bias (D10). Based on the descriptions provided for each domain in SYRCLE’s tool, studies were rated as high risk (+), low risk (−), or unclear (?).

### 2.5 Article selection

Two authors (JK and RP) independently screened the articles retrieved from the four databases. Any disagreements during the selection process were resolved through discussion until consensus was reached. During the title screening stage, articles were filtered for broad relevance; specifically, non-original articles (e.g., reviews, letters, books) and clearly irrelevant subjects (e.g., human clinical trials, cell culture studies) were excluded. The abstract screening stage then focused on identifying studies specifically relevant to METH or AMPH withdrawal and cognition. Finally, the remaining articles underwent a rigorous full-text review, where they were evaluated against the comprehensive inclusion and exclusion criteria to ensure only studies with appropriate control groups and validated cognitive tasks were retained.

## 3 Results

**Figure 1:**
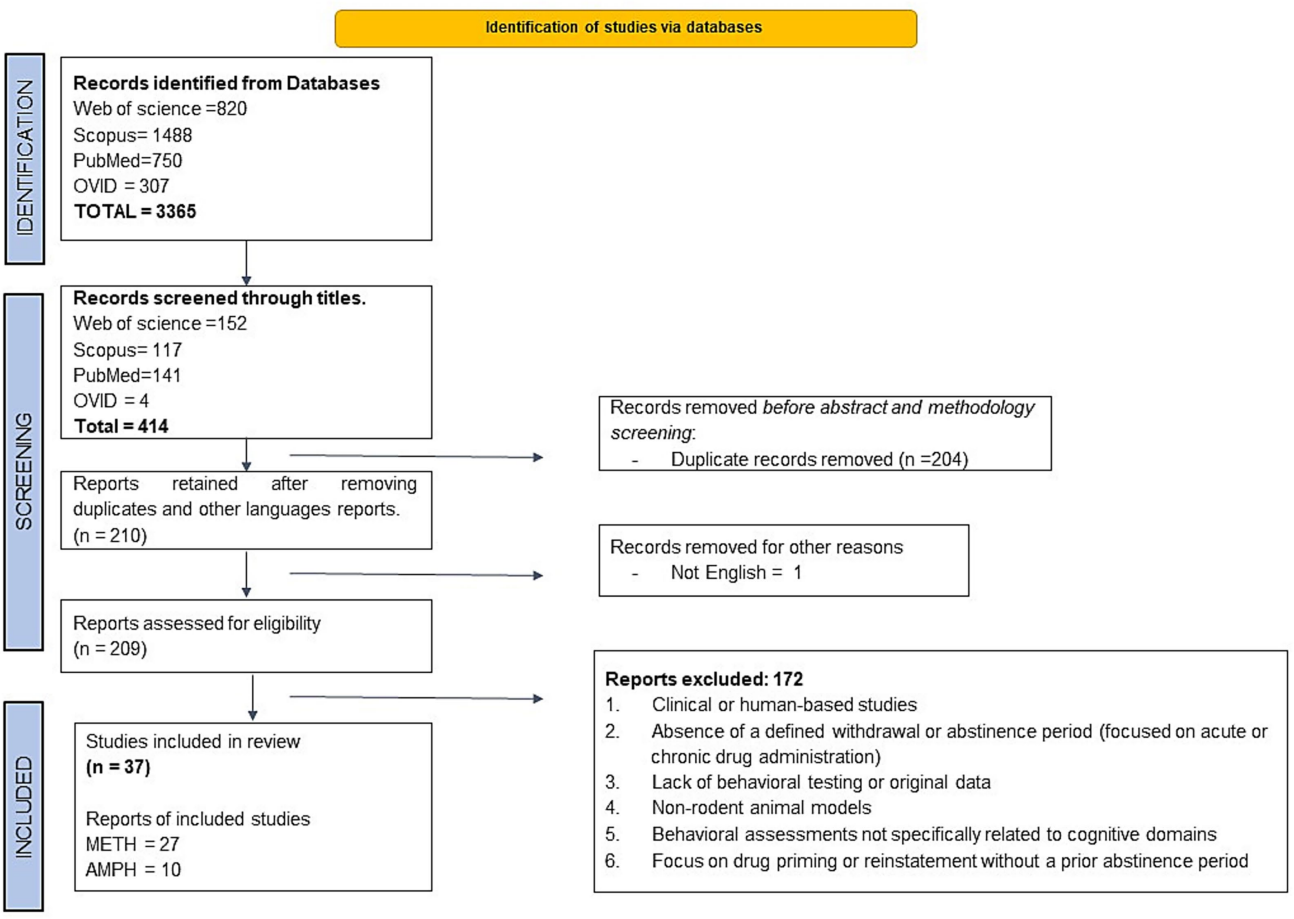
A summary of the literature search, screening, and selection of studies following the Preferred Reporting Items for Systematic Reviews and Meta-Analyses (PRISMA) 2020 guidelines.

The initial keyword search retrieved 3,365 articles (Web of Science = 820, Scopus = 1,488, PubMed = 750, and OVID = 307). Following title screening, 414 articles remained (Web of Science = 152, Scopus = 117, PubMed = 141, and OVID = 4). Subsequently, 204 duplicate articles and one non-English publication were removed. A total of 209 articles were then assessed based on their abstracts, methodology, and results in accordance with the inclusion criteria, resulting in the exclusion of 172 studies. Ultimately, 37 original articles were included in this review (Figure 1).

### 3.1 Characteristics of the studies

**Figure 2:**
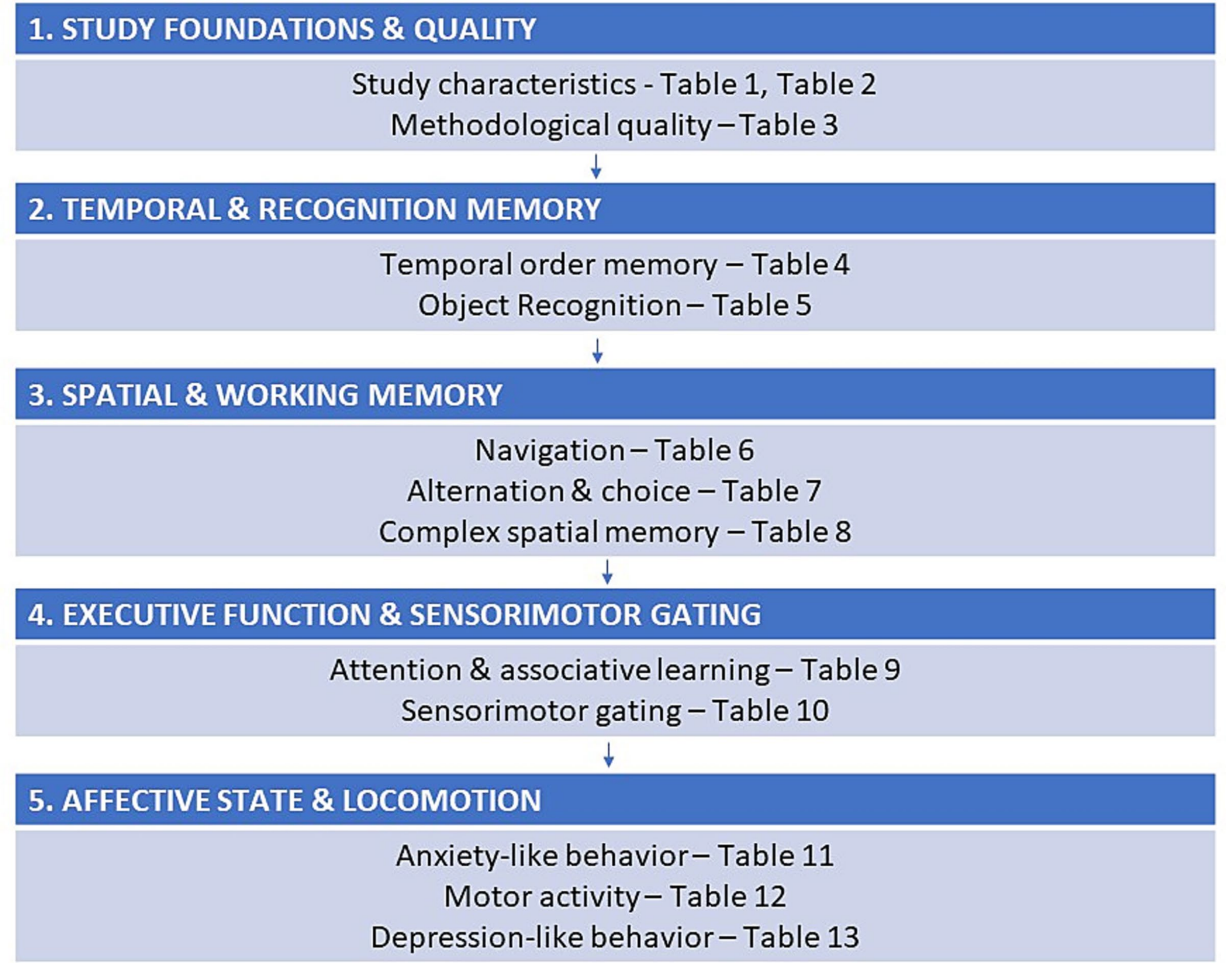
Overview of behavioral test domains with their corresponding tables for detailed data reference.

All 37 included studies investigated behavioral outcomes following METH or AMPH administration in rodents (Figure 2). In each study, animals received METH or AMPH for a defined duration, followed by an abstinence period prior to behavioral testing. Withdrawal periods ranged from 24 h to approximately 1.5 months.

The methodological characteristics of all 37 included studies are integrated into the behavioral outcome tables summarized across Table 1 (METH) and Table 2 (AMPH). All identified research investigated behavioral outcomes following the administration of these stimulants in rodents, where animals received the drug for a defined duration followed by a withdrawal or abstinence period ranging from 24 h to approximately 2 months. As detailed in the tables, the most frequently utilized rat strains included Sprague Dawley, Wistar, Long Evans, and White Norwegian rats, while Fischer and Lewis strains were primarily used for specific genetic comparisons. Among mouse studies, the C57BL/6 strain was the most prevalent, alongside occasional use of CD1 and albino models. While most studies focused on male rodents, several incorporated both sexes to assess dimorphic effects, and a small subset investigated exclusively female populations.

**Table 1:** Characteristics of studies investigating METH exposure.

Reference	Drug	Mode of administration	dosage	Frequency	Period of administration	Behavioral test and outcomes
Kim et al. (2022)	METH	Intraperitoneal injection	(0.5–4.0 mg/kg)	1 to 3 times daily	14 days Day 1 to 3 = 1×/day of 0.5, 1.0, and 1.5 mg/kg. Day 4 to 6 = 2×/day of 1.5, 2.0, and 2.5 mg/kg Day 8 to 10 = 3×/day of 2.5, 3.0, and 3.5 mg/kg Day 11 to 13 = 3×/day of 4.0 mg/kg [No METH on day 7 and 14]	Open Field: No locomotor or anxiety changes Rotarod: Impaired motor coordination Water cross maze: Egocentric navigation bias (spatial strategy shift)
Daberkow et al. (2008)	METH	Injection	10 mg/kg	4 times in 2 h interval	1 day	T-Maze: No significant performance deficit
Recinto et al. (2012)	METH	Intravenous self-administration	0.05 mg/kg	5 days per week	22 days	Y-Maze: LgA rats ↓ spontaneous alternation (working memory deficit) DNMS (T-maze): ↓ % correct responses in LgA group
Reichel et al. (2012)	METH	Self-administration	20 μg (M) and 17.5 μg (F)	1 h or 6 h per day	21 days (7 days for 1 h then half rats given 1 h/day other 6 h/day for 14 days)	Extended-access male and female groups (6 h) ↓ recognition score in OR and OiP
North et al. (2013)	METH	Intraperitoneal injection	24 mg/kg	Once daily	14 days	Novel Object Recognition: No impairment Novel Spatial Recognition: Persistent spatial memory disruption (up to 3 weeks)
Le Cozannet et al. (2013)	METH	Intravenous self-administration	0.05 mg/kg	Extended = 1 h, 3 h and 6 h (5 times) followed by 12 h (20 times) Limited = 1 h (35 times)	63 days 2 days no METH after each 5 times set and 1 day of no METH after each 12 h session	Object Place Recognition: Extended-access failed novel location preference Open Field: Hypolocomotion
Stolyarova et al. (2015)	METH	Injection	0.3 to 6 mg/kg	5 days per week	4 weeks (0.3 mg/kg increment)	Effortful 8-arm Maze: Medium-effort reward preference; no latency differences
Mouton et al. (2016)	METH	Subcutaneous injection	0.2 to 6.0 mg/kg	Twice daily	16 days (0.2 mg/kg increment)	Novel Object Recognition: No novel preference Forced Swim Test: ↓ immobility (PND35); ↑ immobility (PND60) Social Interaction: ↓ social interaction time Open Field: Hypolocomotion at PND35; no change at PND60
Hajheidari et al. (2017)	METH	Subcutaneous injection	2 mg/kg	Twice daily	14 days	Morris Water Maze: ↓ time in target quadrant (spatial memory impairment); acquisition intact
Tran et al. (2018)	METH	Subcutaneous injection	1 mg/kg	Once daily	7 days	WT/non-TG: ↓ Novel object preference
Tu et al. (2019)	METH	Intraperitoneal injection	2 mg/kg	Once daily	5 days	Open Field: no changes Elevated Plus maze: Anxiety Morris Water Maze: ↑ escape latency; ↓ platform crossings
Westbrook et al. (2020)	METH	Intravenous self-administration	0.1 mg/kg	Once daily (2 h ShA and 6 h LgA)	7 days(ShA) then 14 days(LgA)	Object Recognition: No significant differences Object-in-Place: Adolescent-onset METH ↓ novelty exploration
Avila et al. (2021)	METH	Voluntary oral administration	0.25, 0.5 and 1.0 mg/kg	1 or 4 or 16 dose per day	28 days Days 1–3: 1×/day, 0.25 mg/kg Days 4–6: 4×/day, 0.25 mg/kg Days 7–8: 16×/day, 0.25 mg/kg Days 9–10: 16×/day, 0.5 mg/kg Days 11–28 *(Static Phase)*: 16×/day, 1.0 mg/kg	Radial Arm Maze: Female-specific working memory deficits Elevated Plus Maze: Anxiety Tail Suspension Test: No change in immobility
Shi et al. (2021)	METH	Intraperitoneal injection	3 mg/kg	Once daily	10 days	Morris Water Maze: impaired memory Y-Maze: ↓ novel arm preference Object Location Memory: ↓ discrimination index
Armenta-Resendiz et al. (2022)	METH	Intraperitoneal injection	1 mg/kg and 5 mg/kg	Once daily	14 days 1 mg/kg (Day1 and 14) and 5 mg/kg (days 2 to 13)	Temporal Order Memory: Impaired Delayed Non-Match-to-Sample: ↓ % correct choices
Memos et al. (2023)	METH	Voluntary oral administration	0.25, 0.5 and 1.0 mg/kg	1 or 4 or 16 dose per day	28 days Days 1–3: 1×/day, 0.25 mg/kg Days 4–6: 4×/day, 0.25 mg/kg Days 7–8: 16×/day, 0.25 mg/kg Days 9–10: 16×/day, 0.5 mg/kg Days 11–28 *(Static Phase)*: 16×/day, 1.0 mg/kg	Radial 8-arm Maze: No overall deficit; higher female intake linked to impairment
Busceti et al. (2024)	METH	Intraperitoneal injection	1 mg/kg	Once daily	5 days	Novel Object Recognition: ↓ discrimination index (WT) Open Field: No locomotor change
Armenta-Resendiz et al. (2024)	METH	Intraperitoneal injection	1 mg/kg and 5 mg/kg	Once daily	14 days 1 mg/kg (Day1 and 14) and 5 mg/kg (days 2 to 13)	Temporal Order Memory: Impaired WD7; recovery in females WD28
Loxton and Canales (2017)	METH	Oral gavage	2 mg/kg	Once daily	5 days	Open Field: ↓ locomotion Elevated Plus Maze: ↑ anxiety-like behavior Radial Arm Maze: ↑ reference memory errors
Roshani et al. (2022)	METH	Intraperitoneal injection	10 mg/kg	Once daily	5 days	Morris Water Maze: ↓ time in target quadrant; partial recovery at 72 h
Denning et al. (2024)	METH	Oral self-administration	0.8 g/L, 1.6 g/L, or 3.2 g/L (30 mg/kg)	1 h daily (> 10 mg/kg/day)	7 days	Light–Dark: No photophobia effect Novel-Object Reactivity: No neophobia Elevated Plus Maze: No differences Marble Burying: No changes Forced Swim: No significant coping change Morris Water Maze: 1.6 g/L impaired spatial memory Radial Arm Maze: No working/reference memory deficit PPI: No effect
Gao et al. (2025)	METH	Intraperitoneal injection	3 mg/kg	Once daily	7 days	Open Field: ↓ center time (Anxiety) Elevated Plus Maze: ↓ open arm time (Anxiety) Novel Object Recognition: ↓ recognition index (24 h) Y-Maze: ↓ alternation (impaired) Forced Swim: ↑ immobility
Modrak et al. (2025)	METH	Intravenous self-administration	0.32 mg/mL (females)and 0.40 mg/mL (males) [0.1 mg/kg/infusion]	ShA = 1 h daily LgA = 6 h daily	ShA = 7 days followed with LgA = 14 days	Delayed Match-to-Sample: Decrease performance except for 0 delays in both sexes
Mai et al. (2024)	METH	Intraperitoneal injection	3 mg/kg, 0.2 mL	Once daily	10 days	Y-Maze: ↓ novel arm preference Object Location Memory: ↓ discrimination index
Ismail et al. (2025)	METH	Intraperitoneal injection	5 mg/kg	Once daily	21 days	Light–dark compartment test: ↑anxiety-like behavior Elevated Plus Maze: ↑anxiety-like behavior Morris Water Maze: longer latency Forced Swim: ↓ immobility Hole Board: ↑anxiety-like behavior Novel Object Recognition: Impaired Social Interaction: ↑ interaction
Anyanwu et al. (2025)	METH	Intraperitoneal injection	8 mg/kg	Once daily	21 days	Walking Beam: Impaired motor coordination Y-Maze: ↓ alternation (impaired) Novel Object Recognition: ↓ discrimination index
Acuña et al. (2025)	METH	Intravenous self-administration	0.05 mg/kg	96-h sessions /Weekly	3 weeks	Attentional Set Shift Task: Impaired learning/attention in females; reversal intact

**Table 2:** Characteristics of studies investigating AMPH exposure.

Reference	Type of drug	Mode of administration	Dosage	Frequency of administration	Results	Behavioral test and outcomes
Banerjee (1971)	AMPH	Subcutaneous injection	1.5 mg/kg	Once daily	(2nd series) = 3 to 4 weeks (including withdrawal) (3rd series) = 16 days	CAR: ↑ RT; ↑ ER%; CER abolished
Yamamura et al. (1993)	AMPH	Intraperitoneal injection	2 mg/kg	Once daily	20 days	Active/Passive Avoidance: Persistent discrimination deficit
Banerjee (1974)	AMPH	Orally (Dissolved in water)	2 to 4 mg/kg (15—30% increase in dose every succeeding week)	Once daily	12 weeks Weeks 1–7.5: AMPH in increasing dosage weekly Weeks 7.5–10: Drug withdrawal period. Weeks 10–12: Drug resumed at pre-withdrawal dosage	CAR: Slight ↑ latency and ER% Y-Maze: Performance recovery
Banerjee (1975)	AMPH	Orally (Dissolved in water)	2.4 mg/kg (low dose) 4.5 mg/kg (high dose)	Once daily	3 to 5 weeks Initial drug phase: 3–5 weeks Withdrawal phase (drug-free): 2.5 weeks Re-exposure phase: 2.5 weeks	CAR: ↑ latency in high-dose group
Bisagno et al. (2004)	AMPH	Intraperitoneal injection	2.6 mg/kg	Once every 2 days	21 days	NOR: ↓ exploration; no novelty preference OF: Hypolocomotion
Russig et al. (2003)	AMPH	Intraperitoneal injection	1, 2, 3 4 or 5 mg/kg	3 times daily	6 days 1, 2 and 3 mg/kg (day 1), 4, 5 and 5 mg/kg (day 2) and 5 mg/kg(days 4–6)	MWM: No spatial impairment; improved reversal learning
Peleg-Raibstein et al. (2009)	AMPH	Intraperitoneal injection	1 to 5 mg/kg	3 times per week	5 weeks (every week 1 mg/kg dose increases starting from 1 mg/kg)	NOR: Fischer: ↑ novel object preference; Lewis: ↓ novel object preference (Strain-dependent effects) MWM: Improved Fischer performance PPI: No change
Richetto et al. (2013)	AMPH	Intraperitoneal injection	1 mg/kg (Low dose) 2.5 mg/kg (High dose)	Once daily	7 days	LI: High dose impaired tone test PPI: No effect
Bisagno et al. (2003)	AMPH	Intraperitoneal injection	3 mg/kg (M) 2.6 mg/kg and 2.0 mg/kg(last injection) (F)	Once 2 days	20 days (10 injection)	OR/OP: Males: Not impaired (at tested delay) Females: Impaired only at longer delay (4 h) (Sex-dependent recognition effects)
Marchese et al. (2025)	AMPH	Intraperitoneal injection	2.5 mg/kg	Once daily	5 days	Y-Maze: ↓ alternation at room temp Hole board: Altered exploration. (Anxiety)

Regarding drug administration, METH was the primary focus in 27 studies, while 10 studies examined AMPH. For METH protocols, intraperitoneal (i.p.) injection was the dominant route, with standardized daily doses typically ranging from 1 mg/kg to 10 mg/kg, though high-dose protocols reached up to 24 mg/kg. Alternative methods included intravenous self-administration (0.02 to 0.1 mg/kg), subcutaneous injections, and voluntary oral administration using either static or escalating dosage regimens.

For AMPH protocols, the majority of studies utilized i.p. injections with dosages between 1 mg/kg and 5 mg/kg. These administrations often followed standardized daily schedules, though some researchers employed escalating regimens or adjusted dosages based on the sex of the animal. Other reported routes included subcutaneous injections and oral delivery through drinking water.

### 3.2 Risk of bias

The risk of bias for all 37 included studies was assessed using SYRCLE’s tool, with the detailed ratings for each domain provided in Table 3. Across the included studies, most SYRCLE domains were rated as “unclear” due to insufficient reporting, particularly for allocation concealment, blinding, and random housing. Incomplete methodological details contributed to inconsistent quality of reporting. Among the SYRCLE domains, D10 (other sources of bias, 100%), D9 (incomplete outcome data, 89.2%), and D8 (selective outcome reporting, 91.9%) were predominantly rated as low risk. These domains assess potential selective reporting, incomplete data, and other methodological biases not captured by the other nine domains.

**Table 3:** Risk of bias assessment using SYRCLE’s tool.

Type of biasness	D1	D2	D3	D4	D5	D6	D7	D8	D9	D10
Banerjee (1971)	?	–	?	?	?	?	?	–	–	–
Yamamura et al. (1993)	–	+	?	+	+	+	?	–	–	–
Banerjee (1974)	?	+	?	?	?	?	?	–	+	–
Banerjee (1975)	?	–	?	?	?	?	?	–	–	–
Bisagno et al. (2004)	–	–	–	–	–	–	–	–	–	–
Kim et al. (2022)	–	–	–	–	–	–	–	–	–	–
Russig et al. (2003)	?	+	?	?	?	?	?	+	–	–
Daberkow et al. (2008)	?	+	?	?	?	?	?	–	–	–
Peleg-Raibstein et al. (2009)	–	+	?	–	?	?	?	–	–	–
Recinto et al. (2012)	–	–	–	–	–	–	–	–	–	–
Reichel et al. (2012)	?	–	?	?	–	–	–	+	–	–
North et al. (2013)	–	+	?	–	–	–	–	–	+	–
Le Cozannet et al. (2013)	?	–	?	?	?	?	?	–	+	–
Richetto et al. (2013)	?	+	?	?	?	?	?	–	–	–
Stolyarova et al. (2015)	?	+	?	?	?	?	?	–	–	–
Mouton et al. (2016)	+	+	+	+	–	–	–	–	–	–
Hajheidari et al. (2017)	?	+	?	?	?	?	?	–	–	–
Tran et al. (2018)	?	–	?	?	?	?	?	–	–	–
Tu et al. (2019)	–	+	–	?	?	?	?	–	–	–
Bisagno et al. (2003)	?	–	?	?	?	?	?	–	–	–
Westbrook et al. (2020)	?	+	?	?	?	?	?	–	–	–
Avila et al. (2021)	?	+	?	–	–	–	–	–	–	–
Shi et al. (2021)	?	–	?	?	?	?	?	–	–	–
Armenta-Resendiz et al. (2022)	?	+	?	?	?	?	?	–	–	–
Memos et al. (2023)	?	+	?	?	?	?	?	–	–	–
Busceti et al. (2024)	?	+	?	?	?	?	?	–	–	–
Armenta-Resendiz et al. (2024)	?	–	?	?	–	–	–	–	–	–
Loxton and Canales (2017)	?	+	?	?	?	?	?	–	–	–
Roshani et al. (2022)	–	–	–	?	?	?	?	–	–	–
Denning et al. (2024)	?	–	?	?	?	?	?	–	–	–
Marchese et al. (2025)	–	+	?	?	?	?	?	+	+	?
Gao et al. (2025)	–	+	?	?	?	?	?	–	–	–
Modrak et al. (2025)	?	+	?	?	?	?	?	–	–	–
Mai et al. (2024)	?	+	?	?	?	?	?	–	–	–
Ismail et al. (2025)	–	+	?	?	–	–	–	–	–	–
Anyanwu et al. (2025)	–	–	?	?	–	–	–	–	–	–
Acuña et al. (2025)	?	+	?	?	?	?	?	–	–	–

Green – low risk of bias; Red – high risk of bias; Yellow – unclear risk of bias.

However, more than 50% of studies were rated as high risk for the baseline characteristics domain (D2), mainly due to missing key animal characteristics such as weight, sex, or age. Six studies (Yamamura et al., 1993; Mouton et al., 2016; North et al., 2013; Banerjee, 1974; Russig et al., 2003; Marchese et al., 2025) consistently demonstrated high-risk ratings across multiple SYRCLE domains, primarily owing to poor methodological detail and incomplete data reporting. Mouton et al. (2016) was the only study rated high risk for sequence generation (D1) because the authors bred their own rats, and breeding constraints precluded true random allocation into groups.

Importantly, none of the included studies reported additional unmeasured or undisclosed data; any data not explicitly measured were excluded from this review. Overall, although the lack of methodological transparency raises some concerns regarding the reliability of individual findings, the absence of unreported or hidden data reinforces confidence in the overall reliability of the evidence.

### 3.3 Cognitive domain

#### 3.3.1 Working and spatial memory

The experimental parameters and study outcomes for Temporal Order Memory (TOM) are summarized in Table 4. Two studies employed the TOM test to evaluate the effects of METH on working memory in rats. Both studies reported impaired memory in METH-pretreated groups at withdrawal day (WD) 7 (Armenta-Resendiz et al., 2022; Armenta-Resendiz et al., 2024). When TOM was reassessed on WD28, memory recovery was observed in female rats, whereas male rats continued to exhibit persistent impairment (Armenta-Resendiz et al., 2024).

**Table 4:** Temporal order memory (TOM): experimental parameters and study outcomes.

Author (Year)	Withdrawal day(s)	Subjects	Drug	Parameters measured	Main findings/Outcome
Armenta-Resendiz et al. (2022)	7–10 days	Long Evans rats	METH	Exploration time	SAL preferred old object METH explored both equally → memory impairment
Armenta-Resendiz et al. (2024)	7 and 28 days	Long Evans rats (OVX)	METH	TOM ratio (novel vs. old)	WD7: memory impaired in both sexes; WD28: memory restored in females OVX females impaired unless treated with E2

Table 5 provides a detailed overview of test characteristics and results for Object Recognition (OR) and Object Placement (OP) recognition tasks. Studies investigating OR and OP tasks following AMPH administration have reported several notable findings. Short-term AMPH withdrawal (5 days) led to OR impairment (Bisagno et al., 2004). In contrast, long-term AMPH abstinence (48–49 days) produced strain-dependent effects, with impairment observed in Lewis rats but improved OR performance in Fischer rats (Peleg-Raibstein et al., 2009). Sex-specific differences were also evident in OR and OP tasks, which used interval delays to vary task difficulty. In the OR test, AMPH-pretreated males were impaired at both short (2-h) and long (4-h) delays, whereas females exhibited impairment only at the long (4-h) delay or under more challenging conditions. In the OP test, males showed no impairment, while both control and AMPH-pretreated females were impaired at the 1-h delay (Bisagno et al., 2003).

**Table 5:** Object recognition and object place recognition: test characteristics and results.

Author (Year)	Withdrawal day(s)	Subjects	Drug	Exploration parameters measured	Test/Main findings
Bisagno et al. (2004)	5 days	Sprague–Dawley rats	AMPH	Total exploration time (sample and recognition)	OR (sample trial) = ↓ Exploration in pre-treated rats OR (recognition trial) = no novelty preference in AMPH-treated rats.
Peleg-Raibstein et al. (2009)	W48–49	Fischer and Lewis rats	AMPH	Exploration time	OR (Sample phase) = ↓ Exploration for AMPH pre-treated rats. Fischer: ↑ novel object preference; Lewis: ↓ novel object preference
Reichel et al. (2012)	WD7 and WD14	Long–Evans rats	METH	Exploration time, recognition index	Extended-access male and female groups (6 h) ↓ recognition score in OR and OiP
North et al. (2013)	7, 14, 21 days	C57BL/6 J mice	METH	Time exploring novel vs. familiar location	OP = Spatial recognition memory disruption persisted up to 3 weeks No effect in OR
Le Cozannet et al. (2013)	2 days	Sprague–Dawley rats	METH	Exploration time	Sample phase OP = No significant differences in time or location-based exploration between groups. Test phase OP = Extended-access group did not spend more time in new location.
Mouton et al. (2016)	PostND35 and PostND60	FRL/FSL rats	METH	Familiar vs. novel object interaction time	PostND35 and PostND60: ↑ Familiar object interaction and no novel object preference.
Tran et al. (2018)	1, 7, 14, 28 days	WT, KO, TG mice	METH	Preference for novel object	WT/non-TG: ↓ Novel object preference; KO/TG: attenuated impairments
Bisagno et al. (2003)	7 days	Sprague–Dawley rats	AMPH	Time exploring novel vs. familiar object	OR = Males: preference to new object at 1 h delay; Females: preference to new object at 1and 2 h delay but no preference at 4 h-delay OP = male groups (both control and AMPH-treated rats) explored the object in the new location ↑ in the 1-, 2- and 4-h delay trials
Westbrook et al. (2020)	7 and 14 days	Sprague–Dawley rats	METH	Exploration time	OR = No significant differences in study phase; No significant effect shown in METH groups. OP = No significant differences in exploration rate in study phase; adolescent-onset METH rats showed lower novelty exploration.
Shi et al. (2021)	14 days	C57/Bl6 mice (WT, CONi and STAT3i)	METH	Exploration time	OP = METH ↓ discrimination index OP = STAT3i in dCA1 astrocytes ↑ discrimination index compared with CONi group
Busceti et al. (2024)	7 days	WT, mGlu2−/−, mGlu3−/−	METH	Novel vs. familiar object discrimination	OR = ↓ Discrimination index in WT; knockout strains showed resilience
Gao et al. (2025)	48 h	C57BL/6J mice	METH	Exploration time	OR = no changes at 3-h interval but 24-h lower recognition index interval.
Mai et al. (2024)	14 days	Wild-type C57BL/6 mice	METH	Discrimination index	OP = Lower discrimination index (impaired)
Ismail et al. (2025)	14 days	Albino mice	METH	Exploration time	OR = reduced time on the novel object (impairment)
Anyanwu et al. (2025)	14 days	Wistar rats	METH	Discrimination index	OR = Lower discrimination index (impaired)

Similarly, studies using METH reported deficits in both object and spatial domains during the test phase (Reichel et al., 2012; Le Cozannet et al., 2013; Mouton et al., 2016; Tran et al., 2018; Shi et al., 2021; Busceti et al., 2024; Gao et al., 2025; Ismail et al., 2025; Anyanwu et al., 2025), with the exceptions of North et al. (2013) and Westbrook et al. (2020), which observed no changes in OR performance. As seen with AMPH-treated rodents, longer inter-trial intervals (24 h) led to impaired performance, whereas shorter delays (3 h) produced no observable changes. Adolescent-onset METH exposure resulted in greater spatial impairments compared to adult-onset exposure (Westbrook et al., 2020). Self-administration of METH caused recognition deficits in both male and female rodents under extended-access conditions, independent of dosage. Escalating METH doses ranging from 0.2 to 6.0 mg/kg produced long-lasting OR impairments that persisted into extended withdrawal periods, including after postnatal day 60 (PND60), regardless of strain (Mouton et al., 2016). Similarly, high-dose METH exposure (24 mg/kg, i.p.) induced spatial recognition impairments that lasted for 3 weeks in pretreated animals (North et al., 2013).

Characteristics and outcomes for the Morris Water Maze (MWM) and Water Cross Maze (WCM) are presented in Table 6. Four studies reported spatial memory impairments in rodents pretreated with either METH or AMPH using the MWM (Hajheidari et al., 2017; Shi et al., 2021; Roshani et al., 2022; Tu et al., 2019; Denning et al., 2024; Ismail et al., 2025), evidenced by longer escape latencies. No spatial memory deficits were observed during the acquisition or retention phases, although enhanced reversal learning was noted during AMPH withdrawal (Russig et al., 2003). However, Denning et al. (2024) reported slower reversal learning in females at high doses, while males showed no effects. In a strain comparison, Peleg-Raibstein et al. (2009) found no differences between Lewis rats and their saline-treated controls, whereas AMPH-pretreated Fischer rats exhibited improved working memory from trial 1 to trial 2. Roshani et al. (2022) also observed partial recovery at 72 h following impairments detected at 24 h. Notably, one study using the WCM reported that METH abstinence did not impair acquisition of the hidden platform location but induced a significant bias toward an egocentric response strategy (Kim et al., 2022).

**Table 6:** Morris water maze and water cross maze - test parameters and outcomes.

Author (Year)	Test	Withdrawal day(s)	Subjects	Drug	Parameters measured	Main findings/Outcome
Russig et al. (2003)	MWM	2–3 days	Wistar rats	AMPH	Escape latency, swim distance, swim speed	AMPH withdrawal does not impair spatial memory acquisition or retention. Enhanced reversal learning observed during AMPH withdrawal
Peleg-Raibstein et al. (2009)	MWM	W50–55	Fischer and Lewis rats	AMPH	Swim distance	Working memory: Lewis = good performance regardless pre-treatment. Fischer: AMPH-pretreated rats improved from saline group from trial 1 and 2.
Hajheidari et al. (2017)	MWM	1 week	Wistar rats	METH	Escape latency, swim distance, swim speed	METH impaired spatial memory; enriched environment improved memory; swim speed unaffected
Tu et al. (2019)	MWM	7 days	WT, Drd1KO, Drd2KO mice	METH	Escape latency, platform crossing	WT had longer latency than controls; Drd1KO performed worse; fewer platform crossings in METH groups
Shi et al. (2021)	MWM	14 days	C57/Bl6 mice (WT, STAT3i)	METH	Escape latency, time in target quadrant	METH withdrawal impaired memory; STAT3i reversed deficits
Roshani et al. (2022)	MWM	24 h and 72 h	Wistar rats	METH	Time in target quadrant	Probe = METH group: ↓ time in target zone; 72 h withdrawal group showed partial recovery
Denning et al. (2024)	MWM	24 h	C57BL/6 J (B6J) mice	METH	Time in target quadrant, Escape latency,	At 1.6 g/L of METH spatial impairment observed in both sexes. Reversal learning was slower in females only at high dose and males had no effects.
Ismail et al. (2025)	MWM	14 days	Albino mice	METH	Time in target quadrant, Escape latency,	Visible trial = METH mice had longer latency
Kim et al. (2022)	WCM	2 weeks	C57BL/6 J mice	METH	Allocentric/egocentric strategy	In acquisition probe test, METH abstinence significantly biased mice towards utilizing egocentric response strategy during spatial navigation

Another line of evidence comes from the Y-maze spontaneous alternation test, which assesses spatial memory under low-incentive conditions and has been used to investigate withdrawal effects. Previous studies have examined both longer withdrawal periods, 2.5 weeks (Banerjee, 1974), 1 week (Marchese et al., 2025), and 2 weeks (Shi et al., 2021; Anyanwu et al., 2025; Mai et al., 2024) as well as shorter periods of only 2 days (Recinto et al., 2012; Gao et al., 2025). Among these studies, only Banerjee (1974) reported evidence of memory recovery; however, this finding was based on a comparison with hunger-driven rats, which exhibited poor performance in the maze learning paradigm. In contrast, the other six studies, regardless of withdrawal duration, consistently reported persistent memory impairments associated with withdrawal (Shi et al., 2021; Recinto et al., 2012; Marchese et al., 2025; Anyanwu et al., 2025; Mai et al., 2024; Gao et al., 2025).

Table 7 summarizes the outcomes for Y-Maze and T-Maze tests. Armenta-Resendiz et al. (2022) used the Y-maze to perform a modified Delayed Non-Matching to Sample (DNMS) task to assess both reference and working memory. METH administration reduced correct choices for the most recent reward location, indicating memory impairment. Another working memory assessment under high-incentive and cognitively demanding conditions using the DNMS procedure in a T-maze was conducted with METH self-administration by Recinto et al. (2012). Longer access to self-administration was associated with poorer memory performance within 3 days of abstinence. Similarly, Modrak et al. (2025) used a modified DNMS maze and observed declines in performance for both sexes following intravenous self-administration of 0.1 mg/kg/infusion, for both long- and short-access conditions. In contrast, T-maze behavioral testing for spatial memory and reversal learning after METH administration, with an extended abstinence period of nearly 6 weeks, revealed no significant differences (Daberkow et al., 2008).

**Table 7:** Summary of Y- maze and T- maze outcomes.

Author (Year)	Withdrawal day(s)	Subjects	Drug	Test type	Parameters measured	Main findings/Outcome
Banerjee (1974)	2.5 weeks	White Norwegian rats	AMPH	Y-Maze	Escape latency	Significant performance improvement and escape latency ↓; cognitive recovery
Shi et al. (2021)	14 days	C57/Bl6 mice (WT, CONi and STAT3i)	METH	Y maze	The percentage of the time spent in NA	METH withdrawal mice exhibited more preference for the NA than that of CONi-treated METH withdrawal mice.
Recinto et al. (2012)	2	Wistar rats	METH	Y-Maze	Spontaneous alternation %	LgA rats had poorer working memory in short delay.
Anyanwu et al. (2025)	14 days	Wistar rats	METH	Y maze	Spontaneous alternation %	Meth decreases percentage alteration (impaired)
Marchese et al. (2025)	7 days	Wistar Rat	AMPH	Y maze	Spontaneous alternation %	↓ Spontaneous alternations (impaired) at room temperature and no changes in cold temperature.
Gao et al. (2025)	48 h	C57BL/6J mice	METH	Y maze	Spontaneous alternation %	↓ Spontaneous alternations (impaired)
Mai et al. (2024)	14 days	Wild-type C57BL/6 mice	METH	Y-maze	The entries and time in novel arm (NA)	Less preference to NA than Y-maze (Impaired)
Daberkow et al. (2008)	~6 weeks	Sprague–Dawley rats	METH	T-Maze	Trials to reach criterion	No significant performance differences observed
Recinto et al. (2012)	3	Wistar rats	METH	Delayed non-match to sample test T -maze	Trials to criterion, % correct	I-ShA rats outperformed LgA and controls
Armenta-Resendiz et al. (2022)	7 to 10 days	V-Cre transgenic Long Evan rats	METH	Delayed non-match to sample test – Y maze	Percentage of correct choices	Meth has decreased correct choices
Modrak et al. (2025)	5 days	Sprague–Dawley	METH	Delayed non-match to sample	The percentage of correct lever press	Decrease performance except for 0 delays in both sexes

Outcomes for the Radial 8-arm Maze (RAM) are detailed in Table 8. The Radial 8-arm maze (RAM) test revealed sex-specific effects in two studies involving voluntary oral METH administration in mice (Memos et al., 2023; Avila et al., 2021), with females showing greater susceptibility to memory deficits than males. In contrast, a strain comparison in rats indicated that Long Evans rats did not exhibit working memory impairments during withdrawal (Loxton and Canales, 2017). Similarly, Denning et al. (2024) reported no changes in performance following higher-dose oral self-administration (30 mg/kg) in both sexes.

**Table 8:** Radial arm maze outcomes.

Author (Year)	Withdrawal day(s)	Subjects	Drug	Test type	Parameters measured	Main findings/Outcome
Avila et al. (2021)	WD13	C57/Bl6 mice	METH	Radial arm maze	Sequence of arm entries	Females had working memory deficits thru escalation VOMA
Memos et al. (2023)	14 days	C57/Bl6 mice	METH	Radial 8 arm maze	Correct arm visits	Females with higher METH intake had more memory issues, and susceptible to cognitive dysfunction during VOMA withdrawal.
Loxton and Canales (2017)	15 days	Long Evans rats	METH	Radial arm maze	RME, WME	RME impaired; WME unchanged
Denning et al. (2024)	24 h	C57BL/6 J (B6J) mice	METH	Radial arm maze	RME, WME	No changes
Stolyarova et al. (2015)	5–7 days	Long Evans rats	METH	Effortful 8-arm maze	Reward preference, arm order	Preference for medium-effort/reward; no latency effect

#### 3.3.2 Decision making

Outcomes regarding decision-making behaviors are detailed in Table 8. A novel effort-based 8-arm maze revealed that METH-withdrawn rats preferred medium-effort/reward options, whereas saline-treated controls predominantly selected low effort / reward choices (Stolyarova et al., 2015) (Table 8).

#### 3.3.3 Attention and aversive/associative learning

Table 9 provides the experimental parameters and reported outcomes for attention and aversive/associative learning tests. All three studies using the conditioned avoidance response (CAR) paradigm reported impaired performance during the extinction phase under AMPH withdrawal (Banerjee, 1971; Banerjee, 1974; Banerjee, 1975). Although error rates and reaction times increased slightly, Banerjee (1974) noted that the observed extinction effects were not meaningful. Banerjee (1975) specifically reported worse performance at higher AMPH doses.

**Table 9:** Attention and aversive/associative learning tests’ experimental parameters and reported outcome.

Author (Year)	Withdrawal day(s)	Subjects	Drug	Type of test	Parameters measured	Main findings/Outcome
Banerjee (1971)	5 sessions (10 days)	White Norwegian rats	AMPH	CAR	Sessions to acquire CAR, Reaction Time (RT), Error Rate (ER%), Conditioned Emotional Response (CER)	AMPH cessation: CER abolished, ER↑, RT↑; significantly deteriorated performances
Banerjee (1974)	2.5 weeks	White Norwegian rats	AMPH	CAR	Sessions to 100% CAR, Response Latency (RL), Error Rate (ER%)	slight RL↑ and ER%↑, some degradation but not meaningful extinction
Banerjee (1975)	2.5 weeks	White Norwegian rats	AMPH	CAR	Response latency	Some deteriorated in high-dose group; increased latency; RL↑ and ER%↑
Yamamura et al. (1993).	5 sessions (10 days)	Sprague Dawley	METH	Active/Passive avoidance	Runs, immobility and success rate	slightly increased excitation with some persistent disruption of inhibition and, comparatively lasting impairment of discrimination
Richetto et al. (2013)	4 weeks	C57BL/6 mice	AMPH	Latent Inhibition (LI)	% time freezing (conditioning, context, tone tests)	High dose AMPH impaired LI in tone test (dose-dependent); no significant effects in context or conditioning
Peleg-Raibstein et al. (2009)	31 days	Fischer an Lewis rats	AMPH	Pavlovian conditioned freezing	percentage of time freezing	For context freezing, Fischer rats exhibited a substantially lower level of freezing which lasted till the end of the session. Pretreatment had no effect. For conditioned freezing, the interaction stemmed from the presence of an initial increase followed by an extinction profile over time in the Lewis but not in the Fischer rats. Pretreatment had no effect.
Acuña et al. (2025)	4 weeks	Long Evans rats	METH	Attentional set shift task (ASST)	Trial to meet criterion	Impaired learning/attention in females No effect in reversal learning

Additionally, active/passive avoidance was assessed by Yamamura and colleagues, where a repeated daily i.p. regimen of 2 mg/kg METH produced slight recovery but persistent disruption in inhibitory control, indicating mild attention deficits (Yamamura et al., 1993). One study employed the latent inhibition (LI) paradigm using conditioned freezing; Richetto et al. (2013) found that a higher AMPH dose (2.5 mg/kg) abolished LI, whereas a lower dose (1 mg/kg) had no effect.

Across studies using the Pavlovian conditioned freezing paradigm, Lewis rats exhibited higher freezing responses than Fischer rats, while pretreatment had no effect on either contextual or conditioned freezing (Peleg-Raibstein et al., 2009). In a sex-specific finding, Acuña et al. (2025) used the attentional set-shifting task to assess adaptation to new rules and reported impaired learning among female rodents.

#### 3.3.4 Sensorimotor gating

The experimental parameters and outcomes for sensorimotor gating are detailed in Table 10. Both studies utilizing the Prepulse Inhibition (PPI) test reported no significant changes following pretreatment (Peleg-Raibstein et al., 2009; Richetto et al., 2013; Denning et al., 2024). However, strain comparisons indicated that Fischer rats consistently exhibited weaker PPI than Lewis rats (Peleg-Raibstein et al., 2009).

**Table 10:** Prepulse inhibition: experimental parameters and study outcomes.

Author (Year)	Withdrawal day(s)	Subjects	Drug	Test Type	Parameters measured	Main findings/Outcome
Peleg-Raibstein et al. (2009)	W38–39	Fischer and Lewis rats	AMPH	PPI	Startle reactivity magnitude, %PPI	No differences across strains or pretreatment; Fischer rats consistently showed weaker PPI
Richetto et al. (2013)	4 weeks	C57BL/6 mice	AMPH	PPI	% PPI	No significant differences due to drug or age group
Denning et al. (2024)	24 h	C57BL/6 J (B6J) mice	METH	Acoustic startle and PPI	Startle amplitude	No significant effects between sex or METH dose

### 3.4 Emotional, psychomotor, and locomotor domains

#### 3.4.1 Anxiety-like behaviors

Table 11 provides a summary of anxiety-like behavioral outcomes following METH and AMPH exposure. Seven studies assessed anxiety-like behavior using the elevated plus maze (EPM). Increased anxiety-like responses were observed in six METH studies (Avila et al., 2021; Loxton and Canales, 2017; Tu et al., 2019; Denning et al., 2024; Gao et al., 2025; Ismail et al., 2025), whereas the AMPH study with the longest abstinence period (26 days) reported no significant effects (Peleg-Raibstein et al., 2009). The open field (OF) test was employed in four studies, with only Loxton and Canales (2017) and Gao et al. (2025) reporting anxiety-like behavior, while the remaining studies found no significant changes (Kim et al., 2022; Tu et al., 2019).

**Table 11:** Anxiety-like behavior outcomes following METH and AMPH exposure.

Reference	Withdrawal day(s)	Species/Strain	Drug	Test type	Anxiety
Kim et al. (2022)	2 weeks	C57BL/6J mice	METH	OF/arm entries	
Peleg-Raibstein et al. (2009)	26 days	Fischer and Lewis rats	AMPH	EPM	
Avila et al. (2021)	7 days	C57/Bl6 mice	METH	EPM	✓
Loxton and Canales (2017)	15 days	Long Evans rats	METH	OF/arm entries	✓
Loxton and Canales (2017)	15 days	Long Evans rats	METH	EPM	✓
Tu et al. (2019)	7 days	WT, Drd1KO, Drd2KO mice	METH	EPM (distance travelled)	✓
Tu et al. (2019)	7 days	WT, Drd1KO, Drd2KO mice	METH	OF (distance travelled)	
Denning et al. (2024)	24 h	C57BL/6 J (B6J) mice	METH	Light–Dark Shuttle Box (Photophobia)	
Denning et al. (2024)	24 h	C57BL/6 J (B6J) mice	METH	Novel-Object Reactivity (Neophobia)	
Denning et al. (2024)	24 h	C57BL/6 J (B6J) mice	METH	EPM	
Denning et al. (2024)	24 h	C57BL/6 J (B6J) mice	METH	Marble Burying	
Marchese et al. (2025)	7 days	Wistar Rat	AMPH	The hole board test (Holes explored)	✓
Gao et al. (2025)	48 h	C57BL/6 J mice	METH	OF (Center zone)	✓
Gao et al. (2025)	48 h	C57BL/6 J mice	METH	EPM (open arm)	✓
Ismail et al. (2025)	14 days	Albino mice	METH	EPM (open arm)	✓
Ismail et al. (2025)	14 days	Albino mice	METH	Hole board test	✓
Ismail et al. (2025)	14 days	Albino mice	METH	Light–dark compartment test	✓

#### 3.4.2 Locomotion and psychomotor

A summary of locomotor behavioral outcomes is presented in Table 12. Locomotor activity was primarily assessed using the open field test during the withdrawal period. Four studies reported hypolocomotion: three involving METH (Mouton et al., 2016; Loxton and Canales, 2017; Le Cozannet et al., 2013) and one involving AMPH (Bisagno et al., 2004). Among the three studies reporting no change, two used mice (Kim et al., 2022; Busceti et al., 2024) and one used rat (Peleg-Raibstein et al., 2009). Peleg-Raibstein et al. (2009) also found no significant differences between Flinders Sensitive and Resistant Line rats despite their genetic variations. Motor coordination, assessed using the rotarod test, was impaired following a two-week METH withdrawal (Kim et al., 2022).

**Table 12:** Summary of locomotor behavioral outcomes in METH and AMPH studies.

Reference	Withdrawal day(s)	Species/Strain	Drug	Test type	Outcome
Bisagno et al. (2004)	5 days	Sprague–Dawley rats	AMPH	OF	Hypolocomotion
Kim et al. (2022)	2 weeks	C57BL/6J mice	METH	OF	No change
Peleg-Raibstein et al. (2009)	28 days	Fischer and Lewis rats	AMPH	OF	No change
Mouton et al. (2016)	1 and 25 days	Flinders Sensitive and Resistant Line rats	METH	Locomotor Activity/ OF	Hypolocomotion at PND35; no change at PND60. No changes among the different strains.
Loxton and Canales (2017)	15 days	Long Evans rats	METH	OF	Hypolocomotion
Busceti et al. (2024)	7 days	WT, mGlu2−/−, mGlu3−/− mice	METH	OF	No change
Kim et al. (2022)	2 weeks	C57BL/6 J mice	METH	Rotarod	Impaired motor coordination
Le Cozannet et al. (2013)	2 days	Sprague–Dawley rats	METH	Locomotor Activity/ OF	Hypolocomotion
Anyanwu et al. (2025)	14 days	Wistar rats	METH	Walking Beam	Impaired motor coordination

#### 3.4.3 Depression like behavior and social interaction

Reported outcomes for depression-like behavior and social interaction are summarized in Table 13. Depression-like behavior was assessed using the forced swim test (FST) (Mouton et al., 2016; Denning et al., 2024; Gao et al., 2025; Ismail et al., 2025), the tail suspension test (TST) (Avila et al., 2021), and the open field test (OFT) (Loxton and Canales, 2017; Ismail et al., 2025). Increased immobility was reported in both the OFT (Ismail et al., 2025) and FST (Loxton and Canales, 2017; Mouton et al., 2016; Gao et al., 2025). In contrast, Avila et al. (2021) and Denning et al. (2024) found no changes in immobility. Interestingly, Ismail et al. (2025) also observed decreased immobility in the OFT.

**Table 13:** Depression-like behavior: reported outcomes in METH and AMPH withdrawal studies.

Reference	Withdrawal day(s)	Species/Strain	Drug	Test type	Results
Mouton et al. (2016)	1 and 25 days	Flinders sensitive and Resistant line rats (♂)	METH	Forced Swim Test	↓ immobility at PND35; ↑ immobility at PND60
Avila et al. (2021)	5–6 days	C57/Bl6 mice (♂♀)	METH	Tail Suspension Test	No change in immobility
Loxton and Canales (2017)	15 days	Long Evans rats (♂)	METH	Open Field (Immobility measure)	↑ Immobility
Mouton et al. (2016)	1 and 25 days	Flinders sensitive and resistant line rats (♂)	METH	Social Interaction Test	↓ Social interaction
Denning et al. (2024)	24 h	C57BL/6J (B6J) mice	METH	Porsolt Forced-Swim Test	No change in immobility
Gao et al. (2025)	48 h	C57BL/6 J mice	METH	Forced swim test	↑ Immobility
Ismail et al. (2025)	14 days	Albino mice	METH	Open field	↑ Immobility
Ismail et al. (2025)	14 days	Albino mice	METH	Forced swim test	↓ Immobility
Ismail et al. (2025)	14 days	Albino mice	METH	Social interaction test	↑ Social interaction

## 4 Discussion

Emerging evidence from rodent studies demonstrates that repeated exposure to METH and AMPH leads to persistent impairments in learning and memory, underscoring the importance of understanding how these effects are shaped by dose, sex, strain, withdrawal duration, and experimental design. Withdrawal from either METH or AMPH consistently disrupts spatial and nonspatial memory; however, outcomes vary according to biological and methodological variables. Notably, most studies examining episodic-like or nonspatial memory have focused on METH, whereas comparatively fewer have investigated AMPH (Peleg-Raibstein et al., 2009; Bisagno et al., 2004; Bisagno et al., 2003). In AMPH studies, administration was typically intraperitoneal for at least 3 weeks. Although long-term impairments were observed (withdrawal days 48–49), deficits were generally milder than those reported for METH, with some strain-dependent improvements in object recognition and sex-specific preservation of object placement performance. Similarly, spatial paradigms such as the Morris Water Maze do not consistently demonstrate impaired acquisition following AMPH withdrawal.

Experimental design further complicates direct comparisons. Intraperitoneal injection is the most common route of administration; however, AMPH doses rarely exceed 5 mg/kg, whereas METH doses administered via the same route can reach up to 24 mg/kg (North et al., 2013). METH studies more frequently employ escalating-dose regimens, extended-access paradigms, or contingent self-administration, whereas AMPH research predominantly relies on experimenter-administered protocols. Intravenous self-administration of ATS has also produced consistent cognitive impairment (Modrak et al., 2025). Differences in total drug intake, access patterns, contingency, and withdrawal duration likely contribute to the more robust and persistent deficits reported for METH compared with AMPH.

Drug dosage is a critical determinant of cognitive outcome. Lower doses and limited exposure generally preserve or in some cases enhance certain behavioral measures without detectable neurotoxicity. For example, low METH doses (2 mg/kg) produced rewarding effects while maintaining normal neuronal and glial markers (Tu et al., 2019; Tran et al., 2018). In contrast, intermediate doses (1.5–2.5 mg/kg) have been associated with impairments in attention and learning (Banerjee, 1971, 1975; Yamamura et al., 1993). In avoidance paradigms, 2 mg/kg AMPH induced mild deficits that were resolved after withdrawal. Similarly, in the Morris Water Maze, escalating AMPH doses preserved acquisition and enhanced reversal learning, possibly reflecting dopaminergic modulation and reduced proactive interference (Peleg-Raibstein et al., 2009; Russig et al., 2003). These findings parallel human studies showing that low-to-moderate doses of METH administered in controlled clinical settings (5–30 mg) can produce short-term improvements in selected cognitive domains (Cruickshank and Dyer, 2009). Thus, low-to-moderate exposure may modulate cognitive flexibility without inducing overt neurotoxicity.

In contrast, higher dosages and extended-access paradigms more reliably produce long-term cognitive deficits. Escalating binge-like regimens and high-dose protocols (e.g., METH up to 24 mg/kg or repeated high-dose schedules) induce persistent spatial and nonspatial impairments (Mouton et al., 2016; North et al., 2013). Moreover, self-administration under extended-access conditions, particularly over prolonged periods further exacerbates cognitive deficits, suggesting that total drug intake and contingency of administration critically influence withdrawal-related impairments (Le Cozannet et al., 2013; Reichel et al., 2012), with spatial deficits linked to hippocampal CA3 plasticity. High-dose exposure is associated with synaptic and neurochemical alterations in the striatum and hippocampus (Nishioku et al., 1999; Onaivi et al., 2002; Swant et al., 2010). Although behavioral recovery may occur after prolonged abstinence, molecular changes, including reduced BDNF, P2X4, and GABA_A_ receptor expression, as well as persistent Arc mRNA alterations remain detectable (Daberkow et al., 2008; Roshani et al., 2022). These findings indicate that higher exposure levels reveal more robust and enduring neurobiological adaptations.

Overall, METH studies generally report more consistent cognitive impairment than AMPH studies, likely reflecting broader dose ranges, higher maximum doses, and greater use of contingent or extended-access paradigms. In contrast, AMPH effects appear more strain-, sex, and dose-dependent under narrower experimental conditions. Such methodological heterogeneity limits direct comparison between the two psychostimulants.

Sex represents another important biological variable influencing outcomes. Female rodents often consume greater amounts of METH during voluntary administration, potentially related to lower baseline hippocampal GluA1 and GluA2 expression (Memos et al., 2023). These differences are associated with working memory impairments but more substantial recovery following prolonged withdrawal (Avila et al., 2021; Memos et al., 2023). Alterations in hippocampal GluA1, PKMζ, and increased *κ*-opioid receptor expression accompany these behavioral patterns. Female rodents also exhibit higher locomotor activity during testing, possibly reflecting dopaminergic differences (Reichel et al., 2012; Bisagno et al., 2003). TOM deficits observed early in withdrawal may resolve in females at later time points, whereas males tend to show more persistent impairment (Armenta-Resendiz et al., 2024). Reduced GABA transporter expression after prolonged abstinence further suggests that hormonal modulation may contribute to resilience. Nevertheless, some studies report no significant sex differences in recognition tasks, potentially due to small dose variations, group-housing effects, or estrous cycle fluctuations interacting with mGlu2/3 signaling (Westbrook et al., 2020; Busceti et al., 2024). Thus, sex-specific effects remain incompletely characterized.

From a mechanistic perspective, these cognitive impairments involve coordinated dopaminergic, glutamatergic, and GABAergic dysregulation within prefrontal and hippocampal circuits. D1 receptor signaling modulates parvalbumin-positive interneurons in the prefrontal cortex, thereby influencing inhibitory control (Armenta-Resendiz et al., 2022). Psychostimulant-induced adaptations extend beyond dopaminergic neurons and encompass broader neurotoxicity-related processes (Anyanwu et al., 2025). Plastic changes in mGlu2 and mGlu3 receptors further implicate glutamatergic mechanisms (Busceti et al., 2024). Short-term memory retention appears to depend predominantly on GABAergic signaling, whereas longer-term processes involve ERK1/2 pathways (Armenta-Resendiz et al., 2022; Tran et al., 2018). Collectively, these findings highlight circuit-level alterations that may represent potential therapeutic targets.

In addition, METH and AMPH differentially affect locomotion (Kumar et al., 2024), and locomotor alterations are not necessarily correlated with cognitive deficits. Open-field studies report approximately equal incidences of hypolocomotion and no significant changes in activity. Acute locomotor changes largely reflect monoaminergic modulation rather than sustained neurotoxicity (Mouton et al., 2016; Ismail et al., 2025). Withdrawal may also induce anxiogenic-like behavior that reduces exploratory activity (Bisagno et al., 2004). Some studies have reported hypolocomotion during extended withdrawal, potentially influenced by testing conditions such as dark-phase assessment (Loxton and Canales, 2017; Kumar et al., 2024). In contrast, longer open-field durations often reveal no significant locomotor differences (Kim et al., 2022; Peleg-Raibstein et al., 2009). Importantly, hypolocomotion does not appear to be solely attributable to dopaminergic depletion or overt neurotoxicity (Le Cozannet et al., 2013), supporting a mechanistic dissociation between locomotor and cognitive effects. Comparatively, only two of eight studies administered AMPH, limiting interpretation and precluding firm conclusions regarding its locomotor effects. Nevertheless, lower intraperitoneal doses of METH have also been reported to produce no significant locomotor changes (Kim et al., 2022; Busceti et al., 2024).

Finally, future research should directly compare contingent and non-contingent administration under harmonized dosing and access conditions, incorporate larger sample sizes, and systematically align experimental variables. Such efforts would reduce methodological heterogeneity and clarify the differential cognitive and neurobiological effects of METH and AMPH.

## 5 Strength and limitations

This is the first systematic review to focus on both AMPH and METH withdrawal effects on cognitive behaviors in rodents. The review applied no date restrictions; therefore, studies published between 1971 and 2025 were included in the analysis. A recent systematic review by Kumar et al. (2024) examined locomotor changes during METH/AMPH withdrawal in detail, and several other reviews have focused on pharmacological treatments. However, none have specifically addressed cognitive performance during abstinence.

The present systematic review aimed to comprehensively document phenotypic changes observed during the abstinence period and to relate these changes to underlying neurobiological and molecular alterations, including potential mechanistic pathways. Brain regions associated with specific cognitive performance alterations were also considered to further support and contextualize the discussion.

This review has several limitations. Although a broad range of findings was extracted across multiple cognitive domains and behavioral paradigms, only statistically significant results were discussed. In addition, many included studies employed relatively small sample sizes, making behavioral outcomes difficult to interpret due to increased variance. Future reviews could further explore other domains of cognition and emotionality associated with METH and AMPH withdrawal, particularly by incorporating studies with larger sample sizes to enhance interpretability and robustness of conclusions.

## 6 Conclusion

The complex effects of AMPH and METH on cognitive domains are rooted in their impact on various brain regions, including the hippocampus, PFC, striatum, and nucleus accumbens. Findings from this systematic review of rodent studies pertaining to non-spatial and spatial memory disruptions closely align with clinical observations in humans with Methamphetamine Use Disorder (MUD). In drug users, chronic stimulant exposure is linked to significant impairments in executive function, inhibitory control, and episodic memory, reflecting the hippocampal and prefrontal cortex dysregulations highlighted in these rodent models.

However, the translational landscape in humans is significantly more complex due to the prevalence of dual diagnoses. Patients with MUD often present with psychiatric comorbidities, such as mood disorders, which can independently exert negative impacts on cognitive performance in addition to substance use. Furthermore, the cognitive limitations induced by METH or AMPH intake, such as increased impulsivity and poor decision-making, create a feedback loop that hinders recovery. These cognitive deficits are clinically significant as they often lead to poor adherence or response to standard treatment regimens, such as Cognitive Behavioral Therapy (CBT), which requires a baseline level of executive function and inhibitory control that may be compromised during withdrawal. Additionally, findings regarding sex-specific behaviors where females may show higher cognitive impairment echo the clinical need for more inclusive human research, as women have historically been underrepresented in substance use disorder studies (Gunn et al., 2022). By mapping these rodent behavioral phenotypes to the complex clinical profile of human MUD, this review underscores the necessity of targeting specific neurotransmitter interactions within the PFC and hippocampus to develop more effective, sex-specific pharmacological interventions that can support cognitive rehabilitation and improve treatment outcomes.

## Data Availability

The original contributions presented in the study are included in the article/supplementary material, further inquiries can be directed to the corresponding author.
